# Trends in Tobacco Use Among Adolescents by Grade, Sex, and Race, 1991-2019

**DOI:** 10.1001/jamanetworkopen.2020.27465

**Published:** 2020-12-02

**Authors:** Rafael Meza, Evelyn Jimenez-Mendoza, David T. Levy

**Affiliations:** 1 Department of Epidemiology, University of Michigan, Ann Arbor; 2 Department of Oncology, Lombardi Comprehensive Cancer Center, Georgetown University, Washington, DC

## Abstract

**Importance:**

Use of e-cigarettes increased among adolescents between 2011 and 2019. However, whether these changes are affecting patterns of use of other tobacco products, especially cigarettes, remains unclear.

**Objective:**

To examine the long-term and recent trends in cigarette smoking and smokeless tobacco product use among US adolescents by grade (8th, 10th, and 12th), sex (male and female), and race (White and Black).

**Design, Setting, and Participants:**

In this cross-sectional study, joinpoint regression analyses were performed to characterize trends in tobacco product use for key sociodemographic groups, identifying change of trend years (joinpoints). Students in the 8th, 10th, and 12th grades at US secondary schools and high schools who participated in the nationally representative Monitoring the Future survey from January 7, 1991, to June 3, 2019, were evaluated.

**Exposures:**

Cigarette smoking and smokeless tobacco product use during the past 30 days.

**Main Outcomes and Measures:**

Past 30-day and daily prevalence of cigarette smoking and smokeless tobacco product use by year, grade, sex, and race. The prevalence trend segments, change of trend years (joinpoints), and annual percentage change (APC) in prevalence within each trend segment were estimated using joinpoint regression.

**Results:**

Since 1991, 487 335 8th-grade, 447 310 10th-grade, and 424 236 12th-grade students have completed the Monitoring the Future survey (including 663 663 girls and 632 698 boys [those who did not respond to the sex question in the survey were excluded from the sex analyses]). Past 30-day and daily smoking prevalence increased in all groups analyzed from 1991 until 1996 and 1997 and has been decreasing ever since, with more rapid reductions in recent years. For example, daily smoking among 12th-grade boys increased at an APC of 4.9% (95% CI, 3.5%-6.3%) from 1991 to 1998, decreased at an APC of −8.0% (95% CI, –9.3% to –6.7%) from 1998 to 2006, decreased at an APC of −1.6% (95% CI, –4.6% to 1.5%) from 2006 to 2012, and decreased at an APC of −17.4% (95% CI, –19.4% to –15.4%) from 2012 to 2019. Similar results were observed for boys in the 8th grade (5.0% [95% CI, 0.1%-10.2%] for 1991-1996, –8.8% [95% CI, –10.0% to –7.6%] for 1996-2011, and –17.3% [95% CI, –22.2% to –12.0%] for 2011-2019) and 10th grade (7.1% [95% CI, 3.7%-10.7%] for 1991-1997, –11.1% [95% CI, –13.9% to –8.2%] for 1997-2005,–0.7% [95% CI, –5.9% to 4.9%] for 2005-2011, and −17.9 [95%, –21.7% to –13.9%] for 2011-2019), for girls in 8th grade (10.9% [95% CI, 5.0%-17.2%] for 1991-1996 and –10.8% [95% CI, –11.7% to –10.0% for 1996-2019), 10th grade (7.2% [95% CI, 3.9%-10.7%] for 1991-1997, –9.5% [95% CI, –10.5% to –8.6%] for 1997-2012, and –16.3% [95% CI, –21.8% to –10.4%] for 2012-2019), and 12th grade (6.5% [95% CI, 3.6%-9.5%] for 1991-1997, –7.2% [95% CI, –8.1% to –6.3%] for 1997-2012, and –17.5% [95% CI, –21.2% to –13.6%] for 2012-2019). Results were similar, too, for Black adolescents (2015-2019 average annual percentage change: –8.6% [95% CI, –10.3% to –6.8%] for 8th graders; –17.7% [95% CI, –26.3% to –8.2%] for 10th graders; and –18.3% [95% CI, –23.9% to –12.2%] for 12th graders) and White adolescents (2015-2019 average annual percentage change: –17.3% [95% CI, –20.6% to –13.7%] for 8th graders; –16.9% [95% CI, –20.5% to –13.2%] for 10th graders; and –17.0% [95% CI, –20.5% to –13.2%] for 12th graders). Smokeless tobacco was used more variably through 2012, followed by consistent decreases in the past 5 years. For instance, smokeless tobacco use in the past 30 days in 10th-grade boys decreased at an APC of –6.5% (95% CI, –7.5% to –5.4%) from 1991 to 2004, increased at an APC of 3.1% (95% CI, –0.8% to 7.1%) from 2004 to 2012, and decreased at an APC of –11.6% (95% CI, –15.7% to –7.4%) from 2012 to 2019. Similarly, daily smokeless tobacco use in 12th-grade boys decreased at an APC of –3.8% (95% CI, –5.4% to –2.1%) from 1992 to 2005, increased at an APC of 3.1% (95% CI, –0.2% to 6.5%) from 2005 to 2015, and decreased at an APC of –23.0% (95% CI, –33.3% to –11.0%) from 2015 to 2019.

**Conclusions and Relevance:**

This cross-sectional study suggests that, despite the increase in the prevalence of e-cigarette use among adolescents between 2011 and 2019, the prevalence of cigarette and smokeless tobacco use has decreased more rapidly during the same period compared with earlier years.

## Key Points

**Question:**

How have trends in cigarette and smokeless tobacco use among adolescents changed in recent years as e-cigarette use increased?

**Findings:**

In this cross-sectional study of 1 297 362 US adolescents surveyed between 2011 and 2019, past 30-day and daily use of cigarettes and smokeless tobacco decreased more rapidly since 2012 as e-cigarette use began to increase. Smoking and smokeless tobacco use reached historically low levels among adolescents in the US.

**Meaning:**

Despite its recent increase in popularity, e-cigarette use does not seem to be counteracting the decreases in other tobacco use prevalence.

## Teaser

This cross-sectional study examines how trends in cigarette and smokeless tobacco use among adolescents by grade, sex, and race have changed in recent years as e-cigarette use has increased.

## Introduction

Although cigarette smoking has decreased steadily among US youth since the mid-1990s,^1^ the introduction into the market of e-cigarettes has raised concerns about a potential reversal in the steady progress against tobacco consumption in the US and elsewhere. With the introduction of e-cigarettes in the mid-2000s, the increase in use since about 2011, and the more recent rapid increases in use among youth,^2,3,4^ the focus on tobacco control has shifted toward this trend. A particular concern is the potential of e-cigarettes to function as a gateway to cigarette smoking, causing the decrease in cigarette smoking seen in recent years to slow or even reverse course.^5,6,7^ Although considerable attention has been devoted to the increase in vaping among youth, especially in the last 2 years, less attention has been devoted to recent youth smoking and use of other tobacco products, such as smokeless tobacco (chewing tobacco, snuff, snus, and dissolvable tobacco) and cigars.

Previous studies^8,9,10^ have assessed the potential association of e-cigarette use with cigarette smoking trends among youth and the general population, focusing on smoking trends in recent years. However, these studies were based on subjective choices of the period of analysis and relatively crude assessments of trend. In this study, we used joinpoint regression to characterize changing trends in tobacco use among youth. Joinpoint regression is a rigorous method to characterize data trends and objectively identify changes in trends using time course data,^11,12^ It is commonly used in the analysis of cancer incidence and mortality rates^13,14,15,16,17^ and other areas of health^18,19,20,21,22^ and more recently has been applied to tobacco control research.^23,24,25,26^ Using the Monitoring the Future (MTF) survey, we analyzed trends in past 30-day and daily cigarette smoking and smokeless tobacco use from 1991 to 2019 among 8th-, 10th-, and 12th-grade adolescents by sex and race.

## Methods

### MTF Survey Data

In this cross-sectional trend-analysis study, we used data from the MTF survey from January 7, 1991, to June 3, 2019. The MTF survey is a nationally representative annual survey of 8th-, 10th-, and 12th-grade students. The MTF survey collects information on the use of tobacco, including cigarette smoking, smokeless tobacco use, and e-cigarette use; illicit drugs; and alcohol, as well as attitudes and beliefs about these substances. This research was classified as exempt from institutional review board approval by the University of Michigan Institutional Review Board; therefore, patient informed consent was not required. All data were deidentified. The study followed the Strengthening the Reporting of Observational Studies in Epidemiology (STROBE) reporting guideline.

### Demographic Groups

Information on cigarette smoking and smokeless tobacco and the demographic characteristics of the respondents was derived for survey years 1991 to 2019 when available. Individuals were categorized into different demographic groups according to their grade (8th, 10th, or 12th), sex (male or female), and self-reported race (Black or White).

### Tobacco Use Definition

#### Cigarette Use

Survey respondents were asked how frequently they smoked during the past 30 days. Response options included none, less than 1 cigarette per day, 1 to 5 cigarettes per day, about a half pack per day, about 1 pack, about 1½ packs, and 2 packs or more. Consistent with the MTF standard definitions, past 30-day smokers included those reporting smoking any amount (<1 cigarette per day or ≥1 cigarette per day). Daily smokers were defined as those reporting a frequency of 1 to 5 cigarettes per day or more.

#### Smokeless Tobacco Use

Participants were also asked about their use of smokeless tobacco during the past 30 days. Response options included not at all, once or twice, once or twice per week, 3 to 5 times per week, about once a day, and more than once a day. Users of smokeless tobacco in the past 30 days included those reporting use once or twice or more. Daily users included those who indicated that they used smokeless tobacco about once a day or more than once a day. For the 1991 survey, because smokeless tobacco use was only assessed for 8th- and 10th-grade students, we restricted the analyses of 12th-grade students to 1992 to 2019.

#### Tobacco Use Prevalence

Survey sample weights were used to calculate annual tobacco use prevalence and SEs for all products and sociodemographic groups considered. Prevalence calculations were performed in Stata statistical software, version 16 (StataCorp LLC).

### Statistical Analyses

We used the Joinpoint Regression software, version 4.6.0.0^12^ to analyze the trends in the annual prevalence of cigarette smoking and smokeless tobacco use from 1991 to 2019. Joinpoint regression allows for breaking the data into time segments to identify years in which there was a statistically significant change in trend. For each time segment, the analysis estimates the annual percentage change (APC) in prevalence during that period and determines whether the APC is statistically different from zero (no trend). We performed stratified joinpoint regression analyses for all sexes and grades for cigarette and smokeless tobacco use combinations and for races and grades for cigarette use, providing the SE for each annual prevalence estimate. Each tobacco product, sex, and grade group was independently analyzed. The average annual percentage change in prevalence for the period of 2015 to 2019 is also reported for all groups.

## Results

Since 1991, 487 335 8th-grade, 447 310 10th-grade, and 424 236 12th-grade students have completed the MTF survey. Among these students, 663 663 were girls and 632 698 were boys (62 520 participants did not answer the sex question). Overall, 215 147 students reported smoking during the past 30 days and 119 141 reported being daily smokers. In addition, 27 302 respondents reported using smokeless tobacco during the last month and 7398 on a daily basis. The eTable in the Supplement gives the distribution of the MTF (unweighted) sample (1991-2019) by demographic and tobacco use groups; these were also categorized into 5-year calendar-year groups.

### Trends in Cigarette Smoking and Smokeless Tobacco Use by Sex and Grade

**Figure 1.**
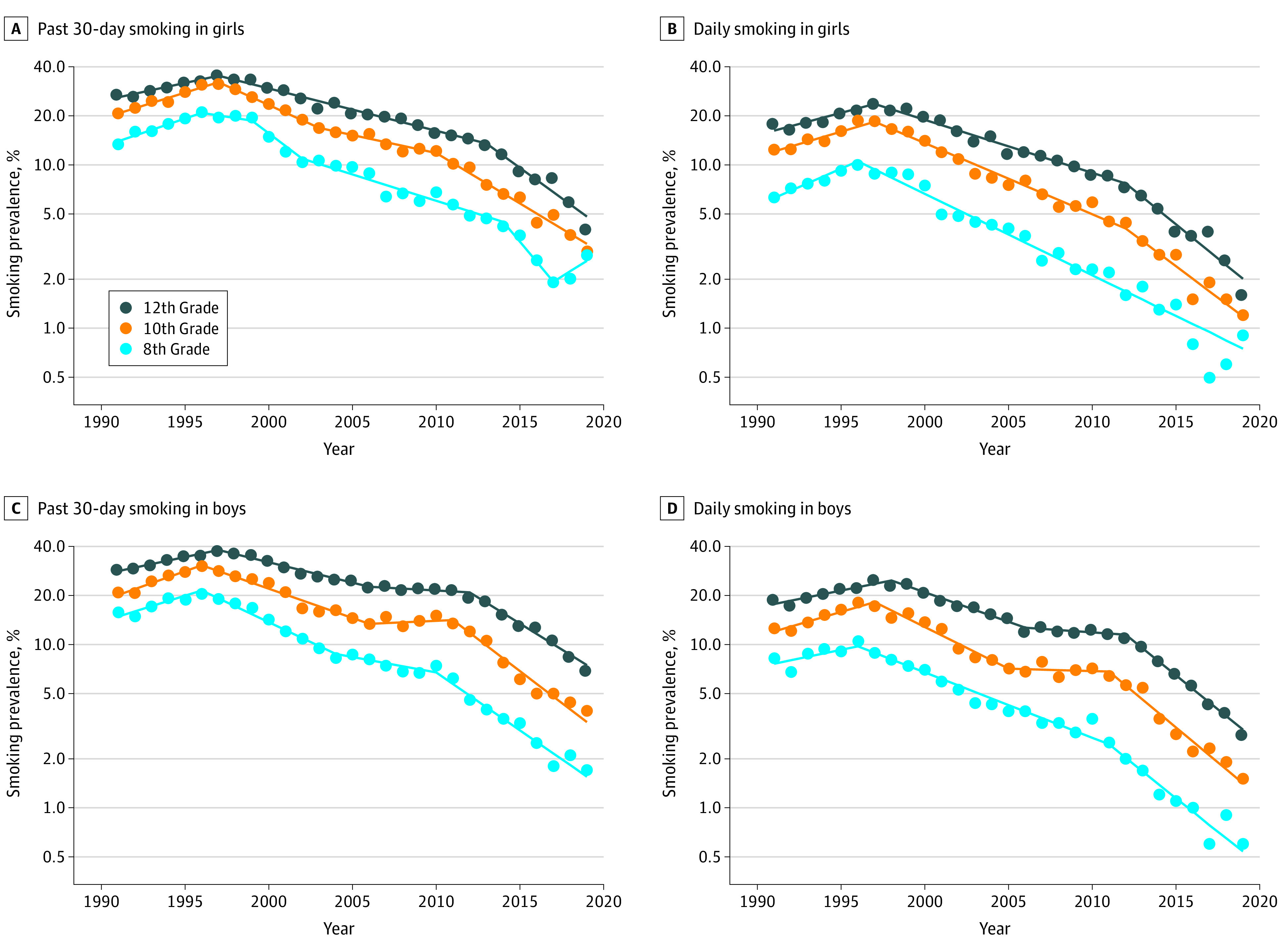
Past 30-Day and Daily Cigarette Smoking Prevalence Among Adolescents by Sex and Grade From 1991 to 2019 The dots represent the annual Monitoring the Future prevalence estimates, and the lines correspond to the best-fitting joinpoint model (Table 1). Data are plotted on a logarithmic scale.

Table 1 gives the tobacco use joinpoint analysis results by sex and grade. Past 30-day and daily smoking prevalence had significant increases in almost every sex and grade category from 1991 until approximately 1996-1998 (trend 1) and decreased thereafter, with a faster decrease in recent years. For instance, past 30-day smoking in 12th-grade girls increased at an APC of 5.2% (95% CI, 2.8%-7.7%) from 1991 to 1997, decreased at an APC of −5.8% (95% CI, –6.4% to –5.2%) from 1997 to 2013, and decreased at a higher APC of −15.8% (95% CI, –19.6% to –11.8%) from 2013 to 2019. Similarly, daily smoking in 12th-grade boys increased at an APC of 4.9% (95% CI, 3.5%-6.3%) from 1991 to 1998, decreased at an APC of –8.0% (95% CI, –9.3% to –6.7%) from 1998 to 2006, decreased at an APC of –1.6% (95% CI, –4.6% to 1.5%) from 2006 to 2012, and decreased at a higher APC of –17.4% (95% CI, –19.4% to –15.4%) from 2012 to 2019. Figure 1 shows the observed (points) daily and past 30-day smoking prevalence by sex and grade (Table 1). Figure 1 illustrates the large decrease in smoking among adolescents since the 1990s, with faster decreases in recent years. For instance, 10th-grade daily smoking decreased at an APC of −16.3% (95% CI, −21.8% to −10.4%) from 2012 to 2019 in girls and at an APC of −17.9% (95% CI, −21.7% to −13.9%) from 2011 to 2019 in boys. No major differences in smoking prevalence rates and trends are observed by sex.

**Table 1.** Trends in Cigarette Smoking and Smokeless Tobacco Use Among Adolescents in the Monitoring the Future Surveys by Grade and Sex

Variable	Trend 1		Trend 2		Trend 3		Trend 4		Trend 5		Trend 6		AAPC, % (95% CI) (2015-2019)
	APC, % (95% CI)	Years	APC, % (95% CI)	Years	APC, % (95% CI)	Years	APC, % (95% CI)	Years	APC, % (95% CI)	Years	APC, % (95% CI)	Years	
**Past 30-d smoking**													
All students													
8th Grade	7.4^a^ (3.7 to 11.2)	1991-1996	–4.8^a^ (–18.8 to 11.7)	1996-1999	–16.2 (–31.3 to 2.1)	1999-2002	–6.1^a^ (–8.4 to –3.6)	2002-2011	–14.2^a^ (–17.6 to –10.6)	2011-2019	NA	NA	–14.2^a^ (–17.6 to –10.6)
10th Grade	6.9^a^ (5.1 to 8.6)	1991-1997	–9.7^a^ (–11.4 to –8.0)	1997-2004	–3.1^a^ (–5.1 to –1.0)	2004-2011	–14.9^a^ (–17.1 to –12.7)	2011-2019	NA	NA	NA	NA	–14.9^a^ (–17.1 to –12.7)
12th Grade	4.2^a^ (3.1 to 5.2)	1991-1998	–7.4^a^ (–9.7 to –5.0)	1998-2003	–4.0^a^ (–4.9 to –3.2)	2003-2013	–15.0^a^ (–17.0 to –12.9)	2013-2019	NA	NA	NA	NA	–15.0^a^ (17.0 to –12.9)
Girls													
8th Grade	8.4^a^ (4.4 to 12.5)	1991-1996	–3.6 (–10.3 to –8.2)	1996-1999	–16.4 (–31.1 to 1.5)	1999-2002	–7.2^a^ (–8.8 to 5.5)	2002-2014	–24.5 (–47.8 to 9.3)	2014-2017	15.8 (–29.5 to 90.4)	2017-2019	–6.5 (–29.2 to 23.6)
10th Grade	7.6^a^ (3.8 to 14.3)	1991-1997	–10.2^a^ (–12.6 to –7.7)	1997-2003	–4.9^a^ (–7.3 to –2.5)	2003-2010	–13.3^a^ (–15.1 to –11.4)	2010-2019	NA	NA	NA	NA	–13.3^a^ (–15.1 to –11.4)
12th Grade	5.2^a^ (2.8 to 7.7)	1991-1997	–5.8^a^ (–6.4 to –5.2)	1997-2013	–15.8^a^ (–19.6 to –11.8)	2013-2019	NA	NA	NA	NA	NA	NA	–15.8^a^ (–19.6 to –11.8)
Boys													
8th Grade	7.7^a^ (4.6 to 10.8)	1991-1996	–10.6^a^ (–12.4 to –8.7)	1996-2004	–4.2 (–8.7 to 0.4)	2004-2010	–15.1^a^ (–17.4 to –12.7)	2010-2019	NA	NA	NA	NA	–15.1^a^ (–17.4 to –12.7)
10th Grade	8.8^a^ (5.0 to 12.7)	1991-1996	–7.9^a^ (–9.3 to –6.6)	1996-2006	1.1 (–4.8 to 7.3)	2006-2011	–16.5^a^ (–19.7 to –13.1)	2011-2019	NA	NA	NA	NA	–16.5^a^ (–19.7 to –13.1)
12th Grade	5.1^a^ (3.7 to 6.5)	1991-1997	–5.6^a^ (–6.4 to –4.8)	1997-2006	–1.3 (–3.6 to 1.0)	2006-2012	–13.6^a^ (–15.3 to –11.9)	2012-2019	NA	NA	NA	NA	–13.6^a^ (–15.3 to 11.9)
**Daily smoking**													
All students													
8th Grade	7.7^a^ (3.3 to 12.4)	1991-1996	–9.5^a^ (–10.5 to –8.4)	1996-2011	–15.1^a^ (–19.3 to –10.7)	2011-2019	NA	NA	NA	NA	NA	NA	–15.1^a^ (–19.3 to –10.7)
10th Grade	7.8^a^ (5.0 to 10.6)	1991-1997	–11.3^a^ (–14.0 to –8.6)	1997-2004	–4.7 (–7.9 to –1.3)	2004-2011	–16.5^a^ (–19.6 to –13.4)	2011-2019	NA	NA	NA	NA	–16.5^a^ (–19.6 to –13.4)
12th Grade	6.1^a^ (3.9 to 8.3)	1991-1997	–6.4^a^ (–7.0 to –5.8)	1997-2013	–17.5^a^ (–20.6 to –14.3)	2013-2019	NA	NA	NA	NA	NA	NA	–17.5^a^ (–20.6 to –14.3)
Girls													
8th Grade	10.9^a^ (5.0 to 17.2)	1991-1996	–10.8^a^ (–11.7 to –10.0)	1996-2019	NA	NA	NA	NA	NA	NA	NA	NA	–10.8^a^ (–11.7 to –10.0)
10th Grade	7.2^a^ (3.9 to 10.7)	1991-1997	–9.5^a^ (–10.5 to –8.6)	1997-2012	–16.3^a^ (–21.8 to –10.4	2012-2019	NA	NA	NA	NA	NA	NA	–16.3^a^ (–21.8 to –10.4)
12th Grade	6.5^a^ (3.6 to 9.5)	1991-1997	–7.2^a^ (–8.1 to –6.3)	1997-2012	–17.5^a^ (–21.2 to –13.6)	2012-2019	NA	NA	NA	NA	NA	NA	–17.5^a^ (–21.2 to –13.6)
Boys													
8th Grade	5.0^a^ (0.1 to 10.2)	1991-1996	–8.8^a^ (–10.0 to –7.6)	1996-2011	–17.3^a^ (–22.2 to –12.0)	2011-2019	NA	NA	NA	NA	NA	NA	–17.3^a^ (–22.2 to –12.0)
10th Grade	7.1^a^ (3.7 to 10.7)	1991-1997	–11.1^a^ (–13.9 to –8.2)	1997-2005	–0.7 (–5.9 to 4.9)	2005-2011	–17.9^a^ (–21.7 to –13.9)	2011-2019	NA	NA	NA	NA	–17.9^a^ (–21.7 to –13.9)
12th Grade	4.9^a^ (3.5 to 6.3)	1991-1998	–8.0^a^ (–9.3 to –6.7)	1998-2006	–1.6 (–4.6 to 1.5)	2006-2012	–17.4^a^ (–19.4 to –15.4)	2012-2019	NA	NA	NA	NA	–17.4^a^ (–19.4 to –15.4)
**Past 30-d smokeless tobacco use**													
All students													
8th Grade	0.1 (–7.0 to 7.6)	1991-1996	–13.9 (–38.0 to 19.5)	1996-1999	–3.0^a^ (–4.1 to –1.9)	1999-2019	NA	NA	NA	NA	NA	NA	–3.0^a^ (–4.1 to –1.9)
10th Grade	–5.7^a^ (–6.9 to –4.4)	1991-2004	4.2 (–0.6 to 9.2)	2004-2011	–9.2^a^ (–12.2 to –6.0)	2011-2019	NA	NA	NA	NA	NA	NA	–9.2^a^ (–12.2 to –6.0)
12th Grade	–5.0^a^ (–6.3 to –3.6)	1992-2004	2.6^a^ (0.0 to 5.3)	2004-2014	–16.2^a^ (–22.5 to –9.4)	2014-2019	NA	NA	NA	NA	NA	NA	–16.2^a^ (–22.5 to –9.4)
Boys													
8th Grade	–5.2^a^ (–5.8 to –4.6)	1991-2019	NA	NA	NA	NA	NA	NA	NA	NA	NA	NA	–5.2^a^ (–5.8 to –4.6)
10th Grade	–6.5^a^ (–7.5 to –5.4)	1991-2004	3.1 (–0.8 to 7.1)	2004-2012	–11.6^a^ (–15.7 to –7.4)	2012-2019	NA	NA	NA	NA	NA	NA	–11.6^a^ (–15.7 to –7.4)
12th Grade	2.5 (–4.9 to 10.5)	1992-1995	–6.5^a^ (–7.9 to –5.0)	1995-2006	15.1 (–6.7 to 41.9)	2006-2009	–3.6^a^ (–6.9 to –0.2)	2009-2016	–20.9^a^ (–31.6 to –8.6)	2016-2019	NA	NA	–16.9^a^ (–24.8 to –8.2)
**Daily smokeless tobacco use**													
All students													
8th Grade	–4.3^a^ (–5.1 to –3.5)	1991-2019	NA	NA	NA	NA	NA	NA	NA	NA	NA	NA	–4.3^a^ (–5.1 to –3.5)
10th Grade	–8.4^a^ (–12.5 to –4.1)	1991-1999	0.5 (–1.9 to 2.9)	1999-2013	–13.9^a^ (–21.5 to –5.6)	2013-2019	NA	NA	NA	NA	NA	NA	–13.9^a^ (–21.5 to –5.6)
12th Grade	–4.9^a^ (–6.9 to –2.8)	1992-2003	2.7^a^ (0.3 to 5.1)	2003-2015	–22.6^a^ (–32.4 to –11.4)	2015-2019	NA	NA	NA	NA	NA	NA	–22.6^a^ (–32.4 to –11.4)
Boys													
8th Grade	–5.0^a^ (–5.9 to –4.2)	1991-2019	NA	NA	NA	NA	NA	NA	NA	NA	NA	NA	–5.0^a^ (–5.9 to –4.2)
10th Grade	–6.7^a^ (–9.4 to –4.0)	1991-2002	1.7 (–2.2 to 5.7)	2002-2013	–15.5^a^ (–23.2 to –7.0)	2013-2019	NA	NA	NA	NA	NA	NA	–15.5^a^ (–23.2 to –7.0)
12th Grade	–3.8^a^ (–5.4 to –2.1)	1992-2005	3.1 (–0.2 to 6.5)	2005-2015	–23.0^a^ (–33.3 to –11.0)	2015-2019	NA	NA	NA	NA	NA	NA	–23.0^a^ (–33.3 to –11.0)

Abbreviations: AAPC, average annual percentage change; APC, annual percentage change; NA, not applicable.

^a^ Significant at *P* < .05.

Because smokeless tobacco prevalence was low in girls, we considered trends only in boys. For both 10th and 12th graders, there was a consistent pattern of decrease in the early years, then a period of increase or flat trend, and finally a period of fast decrease. For instance, smokeless tobacco use in the past 30 days in 10th-grade boys decreased at an APC of –6.5% (95% CI, –7.5% to –5.4%) from 1991 to 2004, increased at an APC of 3.1% (95% CI, –0.8% to 7.1%) from 2004 to 2012, and decreased at an APC of –11.6% (95% CI, –15.7% to –7.4%) from 2012 to 2019. Similarly, daily smokeless tobacco use in 12th-grade boys decreased at an APC of –3.8% (95% CI, –5.4% to –2.1%) from 1992 to 2005, increased at an APC of 3.1% (95% CI, –0.2% to 6.5%) from 2005 to 2015, and decreased at an APC of –23.0% (95% CI, –33.3% to –11.0%) from 2015 to 2019. The eFigure in the Supplement shows the observed (points) and corresponding joinpoint lines of daily and past 30-day smokeless tobacco use prevalence in boys by grade. The eFigure in the Supplement illustrates that, although the decrease in smokeless tobacco use stopped in the early and mid-2000s, it has accelerated in recent years. For instance, daily smokeless tobacco use in 10th-grade boys increased slightly at an APC of 1.7% (95% CI, −2.2% to 5.7%) from 2002 to 2013 but then decreased at an APC of −15.5% (95% CI, −23.2% to −7.0%) from 2013 to 2019.

### Trends in Cigarette Smoking Use by Race and Grade

**Figure 2.**
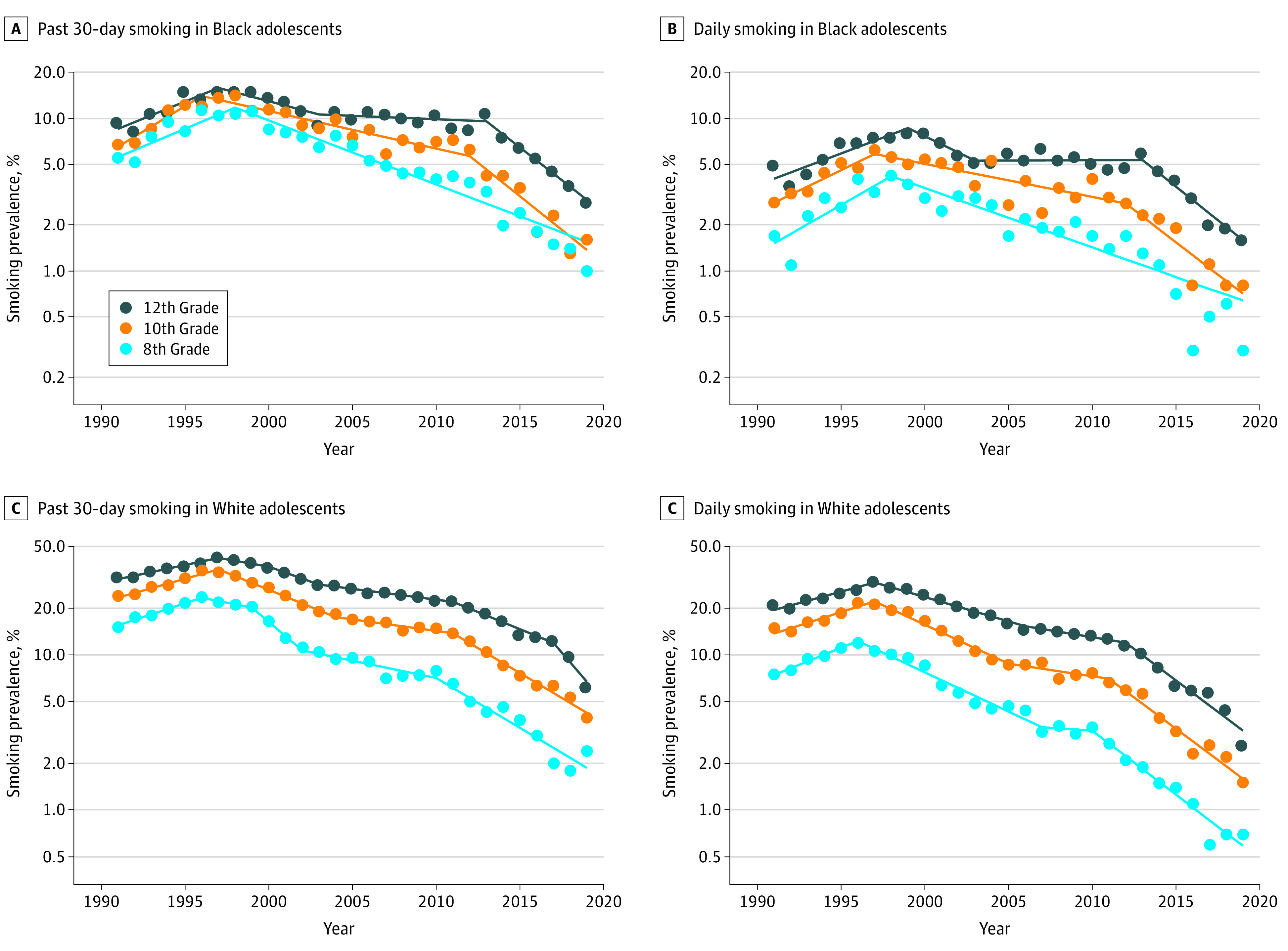
Past 30-Day and Daily Cigarette Smoking Prevalence Among Adolescents by Race (White and Black) and Grade From 1991 to 2019 The dots represent the annual Monitoring the Future prevalence estimates, and the lines correspond to the best-fitting joinpoint model (Table 2). Data are plotted on a logarithmic scale. Notice the different scales between White and Black adolescent smoking prevalence.

Trends in cigarette smoking prevalence by race and grade are presented in Table 2. Patterns of cigarette use by race are consistent with an overall decrease in use for all groups. For the 3 grades and both races, 30-day and daily prevalence of cigarette use increased in the 1990s but continuously decreased thereafter, with faster decreases in recent years. For instance, daily smoking in 10th-grade Black students increased at an APC of 13.1% (95% CI, 3.0%-24.2%) from 1991 to 1997, decreased at a lower APC of −4.9% (95% CI, −6.8% to −2.9%) from 1997 to 2012, and finally decreased at an APC of −17.7% (95% CI, −26.3% to −8.2%) from 2012 to 2019. Similarly, daily smoking in 12th-grade White students increased at an APC of 7.0% (95% CI, 5.1% to 9.0%) from 1991 to 1997, then decreased at an APC of −7.0% (95% CI, –8.2% to –5.7%) from 1997 to 2006, then decreased at a lower APC of −4.0% (95% CI, –7.7% to –0.1%) from 2006 to 2012, and then decreased at an APC of −17.0% (95% CI, –20.5% to –13.2%) from 2012 to 2019. Figure 2 shows the observed (points) daily and past 30-day smoking prevalence by race and grade. Figure 2 also shows that, although smoking among Black adolescents is lower than among White adolescents, both groups have had considerable reductions in smoking, particularly in recent years. For instance, prevalence of daily smoking in 12th-grade White students decreased from 6.3% in 2015 to 2.6% in 2019 (average annual percentage change, −17.0%; 95% CI, −20.5% to −13.2%), whereas in Black students prevalence of daily smoking decreased from 3.9% in 2015 to 1.6% in 2019 (average annual percentage change, −18.3%; 95% CI, −23.9% to −12.2%). Although the analysis did not identify a recent change in trend for smoking among 8th-grade Black adolescents, the observed rates since 2014 are all below the joinpoint model, suggesting that the rate of decrease may have also accelerated for this group. For instance, for past 30-day smoking, the joinpoint model (line) found a prevalence of 1.55% in 2019, but the observed prevalence was 1.0%.

**Table 2.** Trends in Cigarette Smoking Among Adolescents in the Monitoring the Future Surveys by Grade and Race

Variable	Trend 1		Trend 2		Trend 3		Trend 4		Trend 5		Trend 6		AAPC, % (95% CI) (2015-2019)
	APC, % (95% CI)	Years	APC, % (95% CI)	Years	APC, % (95% CI)	Years	APC, % (95% CI)	Years	APC, % (95% CI)	Years	APC, % (95% CI)	Years	
**Past 30-d smoking**													
All students													
8th Grade	7.4^a^ (3.7 to 11.2)	1991-1996	–4.8 (–18.8 to 11.7)	1996-1999	–16.2 (–31.3 to 2.1)	1999–2002	–6.1^a^ (–8.4 to –3.6)	2002-2011	–14.2^a^ (–17.6 to –10.6)	2011-2019	NA	NA	–14.2^a^ (–17.6 to –10.6)
10th Grade	6.8^a^ (4.8 to 8.7)	1991-1997	–9.4^a^ (–11.0 to –7.8)	1997-2005	0.2 (–4.0 to 4.5)	2005-2010	–14.7^a^ (–16.5 to –12.8)	2010-2019	NA	NA	NA	NA	–14.7^a^ (–16.5 to –12.8)
12th Grade	4.2^a^ (3.1 to 5.2)	1991-1998	–7.4^a^ (–9.7 to –5.0)	1998-2003	–4.0^a^ (–4.8 to –3.2)	2003-2013	–15.0^a^ (–17.0 to –13.0)	2013-2019	NA	NA	NA	NA	–15.0^a^ (–17.0 to –13.0)
Black													
8th Grade	11.3^a^ (6.8 to 16.0)	1991-1998	–9.2^a^ (–10.3 to –8.2)	1998-2019	NA	NA	NA	NA	NA	NA	NA	NA	–9.2^a^ (–10.3 to –8.2)
10th Grade	16.6^a^ (8.3 to 25.5)	1991-1996	–5.5^a^ (–6.7 to –4.4)	1996-2012	–18.3^a^ (–25.1 to –10.9)	2012-2019	NA	NA	NA	NA	NA	NA	–18.3^a^ (–25.1 to –10.9)
12th Grade	10.9^a^ (6.7 to 15.2)	1991-1997	–6.5^a^ (–10.7 to –2.0)	1997-2003	–1.0 (–3.3 to 1.2)	2003-2013	–18.0^a^ (–22.4 to –13.3)	2013-2019	NA	NA	NA	NA	–18.0^a^ (–22.4 to –13.3)
White													
8th Grade	8.5^a^ (5.1 to 12.1)	1991-1996	–4.8 (–18.2 to 10.7)	1996-1999	–19.1^a^ (–33.9 to –0.9)	1999-2002	–5.0^a^ (–7.8 to –2.1)	2002-2010	–13.8^a^ (–16.7 to –10.7)	2010-2019	NA	NA	–13.8^a^ (–16.7 to –10.7)
10th Grade	7.2^a^ (5.9 to 8.6)	1991-1997	–9.6^a^ (–11.0 to –8.2)	1997-2004	–3.4^a^ (–5.2 to–1.5)	2004-2011	–13.7^a^ (–15.7 to 11.7)	2011-2019	NA	NA	NA	NA	–13.7^a^ (–15.7 to 11.7)
12th Grade	5.4^a^ (4.4 to 6.4)	1991-1997	–4.2 (–8.9 to 0.6)	1997-2000	–8.5^a^ (–13.9 to –2.7)	2000-2003	–3.1^a^ (–4.1 to –2.2)	2003-2011	–9.7^a^ (–11.8 to –7.5)	2011-2017	–25.1^a^ (–36.5 to –11.6)	2017-2019	–17.7^a^ (–23.7 to –11.3)
**Daily smoking**													
All students													
8th Grade	8.5^a^ (4.0 to 12.9)	1991-1996	–10.2^a^ (–11.9 to –8.5)	1996-2007	0.9 (–23.1 to 27.7)	2007-2010	–17.3^a^ (–20.6 to –13.8)	2010-2019	NA	NA	NA	NA	–17.3^a^ (–20.6 to –13.8)
10th Grade	7.7^a^ (4.9 to 10.7)	1991-1997	–11.2^a^ (–13.5 to –8.9)	1997-2005	–0.7 (–7.0 to 5.9)	2005-2010	–16.6^a^ (–19.2 to –14.0)	2010-1019	NA	NA	NA	NA	–16.6^a^ (–19.2 to –14.0)
12th Grade	6.1^a^ (3.9 to 8.3)	1991-1997	–6.4^a^ (–7.0 to –5.8)	1997-2013	–17.5^a^ (–20.6 to –14.2)	2013-2019	NA	NA	NA	NA	NA	NA	–17.5^a^ (–20.6 to –14.2)
Black													
8th Grade	15.6^a^ (7.4 to 24.4)	1991-1998	–8.6^a^ (–10.3 to –6.8)	1998-2019	NA	NA	NA	NA	NA	NA	NA	NA	–8.6^a^ (–10.3 to –6.8)
10th Grade	13.1^a^ (3.0 to 24.2)	1991-1997	–4.9^a^ (–6.8 to –2.9)	1997-2012	–17.7^a^ (–26.3 to –8.2)	2012-2019	NA	NA	NA	NA	NA	NA	–17.7^a^ (–26.3 to –8.2)
12th Grade	10.0^a^ (6.8 to 13.3)	1991-1999	–11.5 (–23.0 to 1.6)	1999-2003	0.1 (–2.7 to 3.0)	2003-2013	–18.3^a^ (–23.9 to –12.2)	2013-2019	NA	NA	NA	NA	–18.3^a^ (–23.9 to–12.2)
White													
8th Grade	10.6^a^ (6.8 to 14,5)	1991-1996	–11.0^a^ (–12.5 to –9.5)	1996-2007	–1.7 (–24.4 to 28.0)	2007-2010	–17.3^a^ (–20.6 to –13.7)	2010-2019	NA	NA	NA	NA	–17.3^a^ (–20.6 to –13.7)
10th Grade	8.3^a^ (5.8 to 10.9)	1991-1997	–10.9^a^ (–13.0 to –8.7)	1997-2005	–3.7 (–8.3 to 1.2)	2005-2011	–16.9^a^ (–20.5 to –13.2)	2011-2019	NA	NA	NA	NA	–16.9^a^ (–20.5 to –13.2)
12th Grade	7.0^a^ (5.1 to 9.0)	1991-1997	–7.0^a^ (–8.2 to –5.7)	1997-2006	–4.0^a^ (–7.7 to –0.1)	2006-2012	–17.0^a^ (–20.5 to –13.2)	2012-2019	NA	NA	NA	NA	–17.0^a^ (–20.5 to –13.2)

Abbreviations: AAPC, average annual percent change; APC, annual percent change; NA, not applicable.

^a^ Significant at *P* < .05.

## Discussion

In this trend-analysis study, joinpoint regression was used to characterize trends of cigarette smoking and smokeless tobacco use among US adolescents. The focus was primarily on smoking, the greatest cause of disease and death in the US.^27^ The results indicate that downward trends in smoking prevalence slowed in the mid-2000s but then accelerated considerably, reaching rates of decrease by as much as 16% or more per year since approximately 2012. In particular, between 2012 and 2019 for 12th graders, past 30-day smoking decreased from 19.3% to 6.9% for boys and from 14.5% to 4.0% for girls. Daily smoking, a stronger indicator of long-term regular cigarette use,^28^ decreased during the same period from 10.9% to 2.8% for boys and from 7.3% to 1.6% for girls, both at record low levels. Similar trends were observed for White and Black adolescents. These findings are consistent with 2 earlier studies^8,10^ that also found accelerated decreases in smoking since vaping rates increased but are inconsistent with another study.^9^

To our knowledge, this study is the first to consider recent trends in smokeless tobacco use among adolescents. Smokeless tobacco use, although having lower risk than smoking,^27,29^ is also associated with negative health outcomes. Smokeless tobacco use among 10th- and 12th-grade boys decreased between 1990 and 2005 and then increased from 2005 to approximately 2010 for past 30-day use and until approximately 2013 to 2015 for daily use. In 2005, cigarette companies became active in the smokeless tobacco industry and by 2009 controlled more than 80% of the industry and increased marketing and encouraged dual use.^30,31,32^ These actions seem to have resulted in a temporary increase in use; however, as for cigarettes, accelerated decreases in use were seen since approximately 2012.

Although little-cigar use is popular among youth,^33^ data on little-cigar use were not available in the MTF survey until 2014. Although cigar use was decreasing slowly in previous years,^34^ the MTF survey data indicate that regular little-cigar use among 12th graders decreased from 7% in 2014 to 4.9% in 2019, a 30% decrease, whereas flavored cigar use decreased by 35% among 12th graders and 46% among 10th graders.^1^ Thus, little-cigar use has shown patterns of a rapid decrease in recent years, similar to those for cigarette and smokeless tobacco use. Similar patterns have been observed using the National Youth Tobacco Survey.^3^

During the period of greatest decrease in other tobacco products, e-cigarette use increased. Joinpoint analysis of e-cigarette use was not conducted; there is a lack of comparable data from the MTF survey because e-cigarette use was limited before 2011, and definitions in the MTF survey have continued to change.^8,35^ The MTF survey began asking about vaping in 2015,^36^ when past 30-day use was reported at 14.2% for 10th graders and 16.3% for 12th graders. With questions changing in 2016 to ask more broadly about vaporizers, rates decreased to 11.0% for 10th graders and to 12.5% for 12th graders.^37^ With separate questions added about vaporizing nicotine and marijuana in 2017, any vaping reached 13.1% (10th grade) and 16.6% (12th grade) but decreased to 8.2% (10th grade) and 11.0% (12th grade) when confined to nicotine vaping.^36^ The any-vaping rates for 12th graders increased to 26.7% in 2018 and 30.9% in 2019, with these rates being 20.9% in 2018 and 25.5% in 2019 for nicotine vaping.^2^ The National Youth Tobacco Survey was the first youth survey to include questions on vaping.^38^ Past 30-day vaping among high school students increased from 1.5% in 2011 to 4.5% in 2013 and then to 13.4% in 2014,^38^ but only 15.5% of past 30-day vapers report using on 20 or more days of the past 30 days.^39^ Vaping increased slightly to 16.0% in 2015, decreased to 11.7% in 2017, and increased to 20.8% in 2018 and to 27.5% in 2019.^3,40^ Frequent (≥20 days) e-cigarette use among high schoolers increased from 20.0% of e-cigarette users in 2017 to 27.7% in 2018 and then to 34.2% in 2019.^3,40^ Similar patterns were observed in middle school students, with e-cigarette use reaching approximately 5% in 2018.^3^ Therefore, the prevalence of any use and the frequency of e-cigarette use increased in recent years.

Thus, the findings of the present study indicate acceleration of the rates at which cigarette smoking and smokeless tobacco use decreased beginning by 2012 and especially after 2017, when nicotine vaping and the use of Juul and similar vaping products in particular began to accelerate. The association of e-cigarette use with smoking initiation has important implications for public health. Previous analyses^5,41,42,43,44,45^ found that the public health impact of e-cigarette use will largely depend on whether it predominantly acts as a gateway to regular smoking (ie, vaping leads to smoking for those who would not have otherwise smoked, who then become exclusive smokers or dual users of cigarettes and e-cigarettes) or to displace smoking (ie, as vaping prevents smoking for those who otherwise would have smoked and instead vape temporarily or long-term or as a gateway out of smoking for those who previously smoked and instead use e-cigarettes and then quit smoking). The results of the present study do not preclude e-cigarettes acting as a gateway to smoking for some individuals but indicate that such transitions have not been a dominant factor at the population level, at least through 2019. Although some previous studies^7,46,47,48,49^ suggest the importance of such individual transitions from e-cigarette use to smoking, these studies often examined infrequent cigarette use,^7,46,47,48^ examined experimental e-cigarette use,^7,46,47,48^ did not consider the displacement of smoking,^7,46^ and/or did not adequately control for shared risk factors.^49^ The results of the present study indicate that daily smoking, the most concerning form of tobacco consumption, has had particularly large decreases in recent years.

Although the increasingly rapid reductions in cigarette use cannot be attributed to increased e-cigarette use, they closely follow the introduction of e-cigarettes into the market and their increase in prevalence and at least suggest that the recent increase in e-cigarette use has not slowed the decrease in smoking prevalence among youth. Even if the decrease in smoking could be attributed to vaping, these results should not be interpreted to imply that vaping is not a public health concern, particularly at the high rates of youth use observed in 2019.^2,4^ It will be important to monitor e-cigarette use and its use with cigarettes^50^ in future years and consider whether recent increases in e-cigarette use are maintained over time.^51^ At the same time, although the risks of e-cigarette use are uncertain,^52^ if e-cigarettes are of substantially lower risk than cigarettes,^53,54^ then the public health effects of even small reductions in cigarette use are not likely to be offset by larger increases in e-cigarette use.^41,45^

### Limitations

This study has limitations. First, the cross-sectional nature of the MTF survey precludes analyzing individual trajectories of tobacco use among adolescents. Second, the information is self-reported, and the results are subject to recall and response biases. However, school-based surveys have been reported to be reliable for estimation of youth health behaviors.^55^ Third, the MTF survey does not ask specific questions on daily use, and the study consequently relied on questions about average cigarette use per day. Fourth, findings are not generalizable to all youth, including those who are homeschooled or have dropped out of school. Fifth, analyses did not control for other potentially influential variables, such as geographic area or tobacco control policies. Some of the patterns observed can be linked to tobacco policy changes in the US since 1991, but policies that target youth cigarette smoking, such as price and media campaigns, do not appear to have played a major role since 2012.^8^ Although cigarette prices increased substantially in 2009, they have remained relatively stable since then.^56^ The Real Cost media campaign was implemented in 2014 and targeted youth, but studies^57,58^ have found modest associations with youth cigarette use. Thus, tobacco control policies do not appear to explain the major shifts in youth smoking trends observed in recent years.

## Conclusions

In summary, these analyses indicate that past decreases in youth cigarette smoking and smokeless tobacco use continued through 2019 and that these decreases accelerated in recent years since e-cigarette use increased. Thus, despite its recent increase in popularity, e-cigarette use does not seem to be reversing the decreases in other tobacco use prevalence, at least through 2019.

## Notes

Supplement. **eTable.** Sociodemographic Characteristics and Use of Cigarette Smoking and Smokeless Tobacco Use in MTF (1991-2019) **eFigure.** Past 30-Day and Daily Smokeless Tobacco Use Prevalence Among Boys by Grade From 1991 to 2019

## References

[1] Monitoring the Future: a continuing study of American youth (8th- and 10th-grade surveys). Updated September 14, 2020. Accessed June 12, 2020. http://www.monitoringthefuture.org/

[2] Miech R, Johnston L, O’Malley PM, Bachman JG, Patrick ME Trends in adolescent vaping, 2017-2019. N Engl J Med. 2019;381(15):1490-1491. doi:10.1056/NEJMc1910739 31532955 PMC7310772

[3] Gentzke AS, Creamer M, Cullen KA, Vital signs: tobacco product use among middle and high school students—United States, 2011-2018. MMWR Morb Mortal Wkly Rep. 2019;68(6):157-164. doi:10.15585/mmwr.mm6806e1 30763302 PMC6375658

[4] Wang TW, Gentzke AS, Creamer MR, Tobacco product use and associated factors among middle and high school students—United States, 2019. MMWR Surveill Summ. 2019;68(12):1-22. doi:10.15585/mmwr.ss6812a1 31805035 PMC6903396

[5] Stratton K, Kwan LY, Eaton DL, eds. *Public Health Consequences of e-Cigarettes**.* National Academies Press; 2018. 29894118

[6] Chapman S, Bareham D, Maziak W The gateway effect of e-cigarettes: reflections on main criticisms. Nicotine Tob Res. 2019;21(5):695-698. doi:10.1093/ntr/nty067 29660054 PMC6468127

[7] Soneji S, Barrington-Trimis JL, Wills TA, Association between initial use of e-cigarettes and subsequent cigarette smoking among adolescents and young adults: a systematic review and meta-analysis. JAMA Pediatr. 2017;171(8):788-797. doi:10.1001/jamapediatrics.2017.1488 28654986 PMC5656237

[8] Levy DT, Warner KE, Cummings KM, Examining the relationship of vaping to smoking initiation among US youth and young adults: a reality check. Tob Control. 2019;28(6):629-635. doi:10.1136/tobaccocontrol-2018-054446 30459182 PMC6860409

[9] Dutra LM, Glantz SA e-Cigarettes and national adolescent cigarette use: 2004-2014. Pediatrics. 2017;139(2):e20162450. doi:10.1542/peds.2016-2450 28115540 PMC5260150

[10] Foxon F, Selya AS Electronic cigarettes, nicotine use trends and use initiation ages among US adolescents from 1999 to 2018. Addiction. 2020. Published online April 25, 2020. doi:10.1111/add.15099 32335976 PMC7606254

[11] Kim HJ, Fay MP, Feuer EJ, Midthune DN Permutation tests for joinpoint regression with applications to cancer rates. Stat Med. 2000;19(3):335-351. doi:10.1002/(SICI)1097-0258(20000215)19:3<335::AID-SIM336>3.0.CO;2-Z 10649300

[12] Joinpoint Trend Analysis Software. Surveillance Research Program US National Cancer Institute Updated April 22, 2020. Accessed September 20, 2019. https://surveillance.cancer.gov/joinpoint

[13] Ward EM, Sherman RL, Henley SJ, Annual report to the nation on the status of cancer, featuring cancer in men and women age 20-49 years. J Natl Cancer Inst. 2019;111(12):1279-1297. doi:10.1093/jnci/djz106 31145458 PMC6910179

[14] Siegel RL, Miller KD, Jemal A Cancer statistics, 2020. CA Cancer J Clin. 2020;70(1):7-30. doi:10.3322/caac.21590 31912902

[15] Meza R, Meernik C, Jeon J, Cote ML Lung cancer incidence trends by gender, race and histology in the United States, 1973-2010. PLoS One. 2015;10(3):e0121323. doi:10.1371/journal.pone.0121323 25822850 PMC4379166

[16] Salvatore M, Jeon J, Meza R Changing trends in liver cancer incidence by race/ethnicity and sex in the US: 1992-2016. Cancer Causes Control. 2019;30(12):1377-1388. doi:10.1007/s10552-019-01237-4 31606852

[17] Olsen CM, Thompson JF, Pandeya N, Whiteman DC Evaluation of sex-specific incidence of melanoma. JAMA Dermatol. 2020;156(5):553-560. doi:10.1001/jamadermatol.2020.0470 32211827 PMC7097866

[18] Ma J, Ward EM, Siegel RL, Jemal A Temporal trends in mortality in the United States, 1969-2013. JAMA. 2015;314(16):1731-1739. doi:10.1001/jama.2015.12319 26505597

[19] Harding JL, Benoit SR, Gregg EW, Pavkov ME, Perreault L Trends in rates of infections requiring hospitalization among adults with versus without diabetes in the U.S., 2000-2015. Diabetes Care. 2020;43(1):106-116. doi:10.2337/dc19-0653 31615853

[20] Ruch DA, Sheftall AH, Schlagbaum P, Rausch J, Campo JV, Bridge JA Trends in suicide among youth aged 10 to 19 years in the United States, 1975 to 2016. JAMA Netw Open. 2019;2(5):e193886. doi:10.1001/jamanetworkopen.2019.3886 31099867 PMC6537827

[21] Yen EY, Shaheen M, Woo JMP, 46-Year trends in systemic lupus erythematosus mortality in the United States, 1968 to 2013: a nationwide population-based study. Ann Intern Med. 2017;167(11):777-785. doi:10.7326/M17-0102 29086801 PMC6188647

[22] Hobbs FDR, Bankhead C, Mukhtar T, ; National Institute for Health Research School for Primary Care Research Clinical workload in UK primary care: a retrospective analysis of 100 million consultations in England, 2007-14. Lancet. 2016;387(10035):2323-2330. doi:10.1016/S0140-6736(16)00620-6 27059888 PMC4899422

[23] Sawdey MD, Chang JT, Cullen KA, Trends and associations of menthol cigarette smoking among US middle and high school students: National Youth Tobacco Survey, 2011-2018. Nicotine Tob Res. 2020;22(10):1726-1735. doi:10.1093/ntr/ntaa054 32347935 PMC9679736

[24] Kuiper NM, Gammon D, Loomis B, Trends in sales of flavored and menthol tobacco products in the United States during 2011-2015. Nicotine Tob Res. 2018;20(6):698-706. doi:10.1093/ntr/ntx123 28575408 PMC5711620

[25] Tam J e-Cigarette, combustible, and smokeless tobacco product use combinations among youth in the United States, 2014-2019. Addict Behav. 2020;112:106636. doi:10.1016/j.addbeh.2020.106636 32932104

[26] King BA, Gammon DG, Marynak KL, Rogers T Electronic cigarette sales in the United States, 2013-2017. JAMA. 2018;320(13):1379-1380. doi:10.1001/jama.2018.10488 30285167 PMC6233837

[27] National Center for Chronic Disease Prevention and Health Promotion (US) Office on Smoking and Health *The Health Consequences of Smoking—50 Years of Progress: A Report of the Surgeon General.* Centers for Disease Control and Prevention; 2014 Accessed September 20, 2019. https://www.ncbi.nlm.nih.gov/books/NBK179276/24455788

[28] Kozlowski LT, Giovino GA Softening of monthly cigarette use in youth and the need to harden measures in surveillance. Prev Med Rep. 2014;1:53-55. doi:10.1016/j.pmedr.2014.10.003 26844040 PMC4721314

[29] Henley SJ, Thun MJ, Connell C, Calle EE Two large prospective studies of mortality among men who use snuff or chewing tobacco (United States). Cancer Causes Control. 2005;16(4):347-358. doi:10.1007/s10552-004-5519-6 15953977

[30] Levy DT, Yuan Z, Li Y The US SimSmoke tobacco control policy model of smokeless tobacco and cigarette use. BMC Public Health. 2018;18(1):696. doi:10.1186/s12889-018-5597-0 29871597 PMC5989428

[31] Carpenter CM, Connolly GN, Ayo-Yusuf OA, Wayne GF Developing smokeless tobacco products for smokers: an examination of tobacco industry documents. Tob Control. 2009;18(1):54-59. doi:10.1136/tc.2008.026583 18948390

[32] Mejia AB, Ling PM Tobacco industry consumer research on smokeless tobacco users and product development. Am J Public Health. 2010;100(1):78-87. doi:10.2105/AJPH.2008.152603 19910355 PMC2791252

[33] King BA, Tynan MA, Dube SR, Arrazola R Flavored-little-cigar and flavored-cigarette use among U.S. middle and high school students. J Adolesc Health. 2014;54(1):40-46. doi:10.1016/j.jadohealth.2013.07.033 24161587 PMC4572463

[34] Messer K, White MM, Strong DR, Trends in use of little cigars or cigarillos and cigarettes among U.S. smokers, 2002-2011. Nicotine Tob Res. 2015;17(5):515-523. doi:10.1093/ntr/ntu179 25239955 PMC4402355

[35] Levy DT, Yuan Z, Li Y, Mays D, Sanchez-Romero LM An examination of the variation in estimates of e-cigarette prevalence among U.S. adults. Int J Environ Res Public Health. 2019;16(17):E3164. doi:10.3390/ijerph16173164 31480240 PMC6747488

[36] Miech RA, Johnston LD, O’Malley PM, Bachman JG, Schulenberg JE, Patrick ME *Monitoring the Future National Survey Results on Drug Use, 1975-2019: Volume I, Secondary School Students.* University of Michigan Institute of Social Research; 2020 Accessed July 7, 2020. http://www.monitoringthefuture.org/pubs/monographs/mtf-vol1_2019.pdf

[37] Boyd CJ, Veliz P, Evans-Polce RJ, Eisman AB, Esteban McCabe S Why are national estimates so different? a comparison of youth e-cigarette use and cigarette smoking in the MTF and PATH surveys. J Stud Alcohol Drugs. 2020;81(4):497-504. doi:10.15288/jsad.2020.81.497 32800087 PMC7437551

[38] Jamal A, Gentzke A, Hu SS, Tobacco use among middle and high school students - United States, 2011-2016. MMWR Morb Mortal Wkly Rep. 2017;66(23):597-603. doi:10.15585/mmwr.mm6623a1 28617771 PMC5657845

[39] Neff LJ, Arrazola RA, Caraballo RS, Frequency of tobacco use among middle and high school students–United States, 2014. MMWR Morb Mortal Wkly Rep. 2015;64(38):1061-1065. doi:10.15585/mmwr.mm6438a1 26422781

[40] Cullen KA, Gentzke AS, Sawdey MD, e-Cigarette use among youth in the united states, 2019. JAMA. 2019;322(21):2095-2103. doi:10.1001/jama.2019.18387 31688912 PMC6865299

[41] Levy DT, Borland R, Villanti AC, The application of a decision-theoretic model to estimate the public health impact of vaporized nicotine product initiation in the United States. Nicotine Tob Res. 2017;19(2):149-159. doi:10.1093/ntr/ntw158 27613952 PMC5234365

[42] Levy DT, Cummings KM, Villanti AC, A framework for evaluating the public health impact of e-cigarettes and other vaporized nicotine products. Addiction. 2017;112(1):8-17. doi:10.1111/add.13394 27109256 PMC5079857

[43] Levy DT, Fong GT, Cummings KM, The need for a comprehensive framework. Addiction. 2017;112(1):22-24. doi:10.1111/add.13600 27936507 PMC5396387

[44] Cherng ST, Tam J, Christine PJ, Meza R Modeling the effects of e-cigarettes on smoking behavior: implications for future adult smoking prevalence. Epidemiology. 2016;27(6):819-826. doi:10.1097/EDE.0000000000000497 27093020 PMC5039081

[45] Mendez D, Warner KE A magic bullet? the potential impact of e-cigarettes on the toll of cigarette smoking. Nicotine Tob Res. Published online August 21, 2020. doi:10.1093/ntr/ntaa160 32823272 PMC7976928

[46] Khouja JN, Suddell SF, Peters SE, Taylor AE, Munafò MR Is e-cigarette use in non-smoking young adults associated with later smoking? a systematic review and meta-analysis. Tob Control. Published online March 10, 2020. doi:10.1136/tobaccocontrol-2019-055433 32156694 PMC7803902

[47] Villanti AC, Pearson JL, Glasser AM, Frequency of youth e-cigarette and tobacco use patterns in the United States: measurement precision is critical to inform public health. Nicotine Tob Res. 2017;19(11):1345-1350. doi:10.1093/ntr/ntw388 28013271 PMC5896511

[48] Shahab L, Beard E, Brown J Association of initial e-cigarette and other tobacco product use with subsequent cigarette smoking in adolescents: a cross-sectional, matched control study. Tob Control. Published online March 17, 2020. doi:10.1136/tobaccocontrol-2019-055283 32184339 PMC7907552

[49] Kim S, Selya AS The relationship between electronic cigarette use and conventional cigarette smoking is largely attributable to shared risk factors. Nicotine Tob Res. 2020;22(7):1123-1130. doi:10.1093/ntr/ntz157 31680169 PMC7291806

[50] Glasser AM, Johnson AL, Niaura RS, Abrams DB, Pearson JL Youth vaping and tobacco use in context in the United States: results from the 2018 National Youth Tobacco Survey. Nicotine Tob Res. Published online January 13, 2020. doi:10.1093/ntr/ntaa010 31930295

[51] Hair EC, Romberg AR, Niaura R, Longitudinal tobacco use transitions among adolescents and young adults: 2014-2016. Nicotine Tob Res. 2019;21(4):458-468. doi:10.1093/ntr/ntx285 29452385

[52] Eissenberg T, Bhatnagar A, Chapman S, Jordt S-E, Shihadeh A, Soule EK Invalidity of an oft-cited estimate of the relative harms of electronic cigarettes. Am J Public Health. 2020;110(2):161-162. doi:10.2105/AJPH.2019.305424 31913680 PMC6951374

[53] McNeill A, Brose LS, Calder R, Bauld L, Robson D, Public Health England Vaping in England: an evidence update including mental health and pregnancy, March 2020. Accessed June 17, 2020. https://assets.publishing.service.gov.uk/government/uploads/system/uploads/attachment_data/file/869401/Vaping_in_England_evidence_update_March_2020.pdf

[54] Goniewicz ML, Smith DM, Edwards KC, Comparison of nicotine and toxicant exposure in users of electronic cigarettes and combustible cigarettes. JAMA Netw Open. 2018;1(8):e185937. doi:10.1001/jamanetworkopen.2018.5937 30646298 PMC6324349

[55] O’Malley PM, Johnston LD, Bachman JG, Schulenberg J A comparison of confidential versus anonymous survey procedures: effects on reporting of drug use and related attitudes and beliefs in a national study of students. J Drug Issues. 2000;30(1):35-54. doi:10.1177/002204260003000103

[56] Centers for Disease Control and Prevention The tax burden on tobacco, 1970-2018: chronic disease and health promotion data and indicators. Updated August 13, 2020. Accessed September 28, 2020. https://chronicdata.cdc.gov/Policy/The-Tax-Burden-on-Tobacco-1970-2018/7nwe-3aj9

[57] Duke JC, MacMonegle AJ, Nonnemaker JM, Impact of the Real Cost media campaign on youth smoking initiation. Am J Prev Med. 2019;57(5):645-651. doi:10.1016/j.amepre.2019.06.011 31443954 PMC13389946

[58] Farrelly MC, Duke JC, Nonnemaker J, Association between the Real Cost media campaign and smoking initiation among youths - United States, 2014-2016. MMWR Morb Mortal Wkly Rep. 2017;66(2):47-50. doi:10.15585/mmwr.mm6602a2 28103214 PMC5657653

