# Supplementary material for: Trends in Tobacco Use Among Adolescents by Grade, Sex, and Race, 1991-2019
Source: JAMA Netw Open. 2020 Dec 2;3(12):e2027465. doi: 10.1001/jamanetworkopen.2020.27465 (PMC7711321; doi:10.1001/jamanetworkopen.2020.27465)
Supplement: Supplement. — eTable. Sociodemographic Characteristics and Use of Cigarette Smoking and Smokeless Tobacco Use in MTF (1991-2019) eFigure. Past 30-Day and Daily Smokeless Tobacco Use Prevalence Among Boys by Grade From 1991 to 2019 [file jamanetwopen-e2027465-s001.pdf]

Supplementary Online Content

Meza R, Jimenez-Mendoza E, Levy DT. Trends in tobacco use among adolescents by grade, sex, and race, 1991-2019. *JAMA Netw Open*. 2020;3(12):e2027465. doi:10.1001/jamanetworkopen.2020.27465

eTable. Sociodemographic Characteristics and Use of Cigarette Smoking and Smokeless Tobacco Use in MTF (1991-2019)

eFigure. Past 30-Day and Daily Smokeless Tobacco Use Prevalence Among Boys by Grade From 1991 to 2019

This supplementary material has been provided by the authors to give readers additional information about their work.

© 2020 Meza R et al. *JAMA Network Open.*

eTable 1. Sociodemographic characteristics and use of cigarette smoking and smokeless tobacco use in the Monitoring the Future (MTF) surveys 1991-2019

	1991-2019	1991-1995	1996-2000	2001-2005	2006-2010	2011-2015	2016-2019
Grade							
8th	487,335	91,316	90,699	83,939	81,052	77,617	62,712
10th	447,310	78,874	75,531	78,763	80,442	73,560	60,140
12th	424,236	80,302	73,908	72,648	73,918	69,123	54,337
Sex							
Girls	663,663	123,546	119,126	116,539	115,680	105,927	82,845
Boys	632,698	117,891	110,821	108,851	109,755	103,850	81,530
Race							
White	775,204	159,172	144,441	140,411	134,277	115,681	81,222
Black	169,447	33,026	32,214	29,941	28,759	25,676	19,831
Past 30-day smoking							
Yes	215,147	56,744	60,655	39,001	31,405	20,135	8,007
No	1,102,893	187,702	173,636	190,212	197,403	193,320	160,620
Daily smoking							
Yes	119,141	32,871	35,550	21,697	16,256	9,513	3,254
No	1,199,699	211,575	198,741	207,526	212,552	203,942	165,373
Past 30-day smokeless tobacco use							
Yes	27,302	7,226	5,445	4,127	4,475	4,021	2,008
No	467,042	81,078	81,247	82,810	83,002	77,490	61,415
Daily smokeless tobacco use							
Yes	7,398	1,914	1,367	1,160	1,250	1,192	515
No	486,946	86,390	85,325	85,777	86,227	80,319	62,908

© 2020 Meza R et al. *JAMA Network Open.*

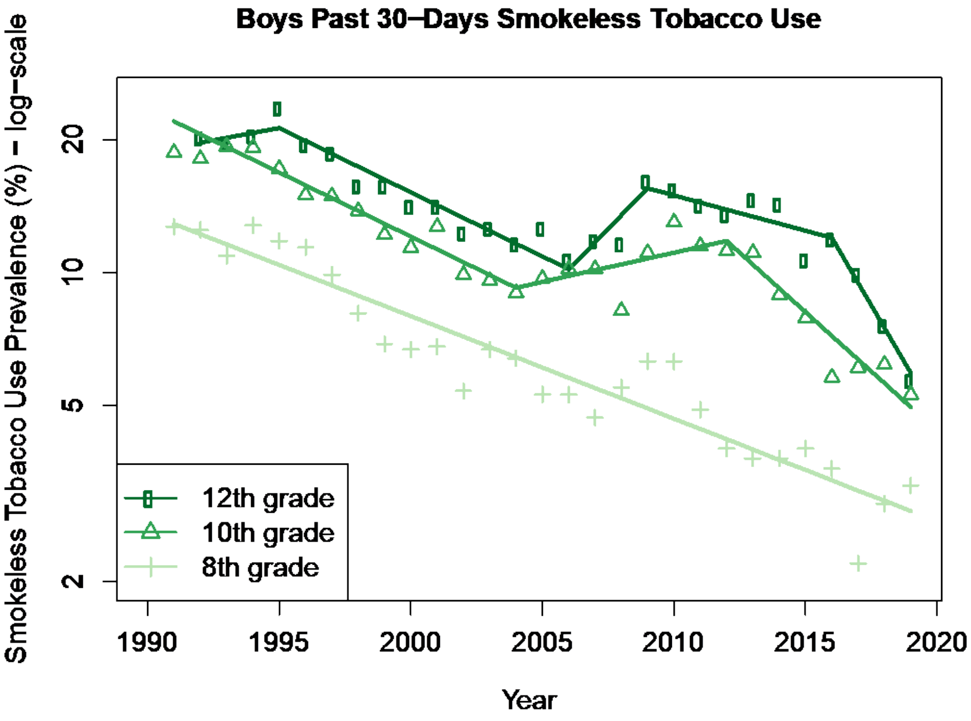

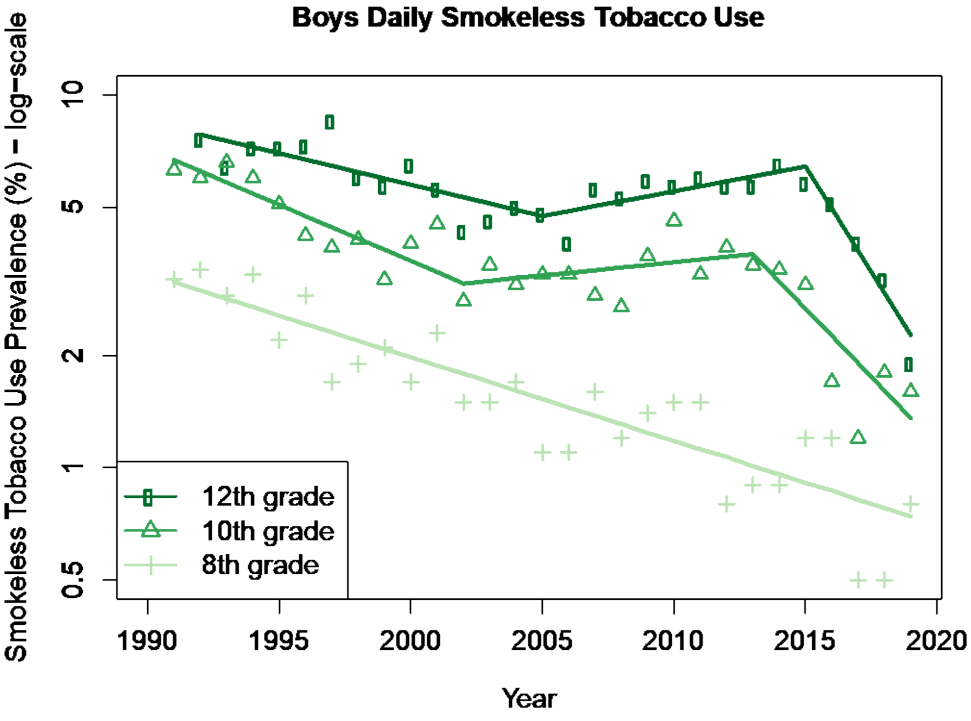

eFigure 1. Past 30-day and daily smokeless tobacco use prevalence among male adolescents by grade from 1991 to 2019 (12th grade data started in 1992). The dots represent the annual MTF prevalence estimates and the lines correspond to the best fitting joinpoint model (see table 1). Logarithmic-scale.

© 2020 Meza R et al. *JAMA Network Open.*
